# Diagrams

**Published:** 1872-03

**Authors:** 


We have received from J. M. Toner, M. D., of Washington, D. C., several diagrams illustrating,

1. The proportion of males to females of the same age in the U. S. from the census of 1860.

2. The ages at which an excess of one sex over the other exists in the different states and territories of the U. S. from the census of 1860.

3. The proportion of white children under 15 years, to the 1000 females between 15 and 40, from 1800–1870. To show the fact of the decline of the birth-rate in the U. S.

IV. The aggregate mortality in the U. S. for the year ending June 1st, 1860·

These diagrams are carefully constructed, and serve to show at a glance the several facts which they are intended to represent.

